# Cutaneous disseminated sporotrichosis associated with diabetes: A case report and literature review

**DOI:** 10.1371/journal.pntd.0011647

**Published:** 2023-09-18

**Authors:** Xiujiao Xia, Huilin Zhi, Zehu Liu

**Affiliations:** Department of Dermatology, Hangzhou Third People’s Hospital, Affiliated Hangzhou Dermatology Hospital, Zhejiang University School of Medicine, Hangzhou, China; Albert Einstein College of Medicine, UNITED STATES

## Abstract

**Background:**

Cutaneous disseminated sporotrichosis (CDS), also called hematogenous sporotrichosis, is a rare condition that usually affects immunocompromised patients. The current work presents the case of a woman with diabetes mellitus associated with CDS.

**Case presentation:**

A 59-year-old woman with diabetes mellitus presented with a two-year history of ulcerated rashes on the left ankle and both sides of the jaw. Physical examination revealed three annular areas of erythematous and raised plaque with an ulcer over the left ankle and both sides of the jaw. Based on laboratory findings, elevated blood glucose concentration and decreased white cell count were observed. *Sporothrix globosa* was identified in the mycological culture of biopsied tissue from the three lesions and this was confirmed by DNA sequencing. The skin lesions healed after two-month itraconazole therapy.

**Conclusions:**

Diabetes is a risk factor for disseminated sporotrichosis, which may be induced by hematogeneous spread, repeated inoculation, or autoinoculation. This study raises awareness among clinicians, with regard to the notion that people with possibly altered immune function are potentially vulnerable to severe clinical forms of sporotrichosis.

## Author summary

Sporotrichosis is one of the unspecified deep mycoses that match the neglected tropical diseases (NTDs) selection criteria. It is caused by *Sporothrix*, a fungus that usually results in zoonotic fungal diseases and sapronosis that are endemic in temperate regions. Sporotrichosis is clinically presented in two main forms, which are lymphocutanous sporotrichosis (LC) and fixed sporotrichosis (F), especially in immunocompetent patients. However, severe clinical versions, such as cutaneous disseminated, mucosal, and extracutaneous forms may present in immunocompromised hosts. This study presents a case of cutaneous disseminated sporotrichosis in a patient with diabetes mellitus and information based on the review of relevant literature. Clinicians should be on the lookout for severe clinical forms of sporotrichosis in all patients with possibly altered immune function.

## Introduction

Sporotrichosis is a subacute or chronic implantation (formerly subcutaneous) fungal infection, related to dimorphic fungi that fall within the *Sporothrix schenckii* complex [1–4]. In humans, the infection is prompted by traumatic inoculation from contaminated sources, such as wood, thorns, and splinters. This explains why it is known as "gardener’s disease." The infection can also be transmitted through scratches and bites from infected cats [5].

Based on the location of the lesions, sporotrichosis can be classified into cutaneous, mucosal, and extracutaneous forms. The cutaneous form is more common [6]. Clinically, the infection is mainly presented as lymphocutanous sporotrichosis (LC) or fixed sporotrichosis (F). Cutaneous disseminated sporotrichosis (CDS) has also been reported, although it is less common [7]. CDS is also called hematogenous sporotrichosis and even though it is rare, more incidences have been reported in immunocompromised patients [4]. CDS is characterized by multiple skin lesions at non-adjacent sites, without extracutaneous involvement. This report presents the case of a woman with diabetes mellitus, who suffered from a *S*. *globosa* infection that disseminated to the jaw and ankle.

### Case report

**Fig 1:**
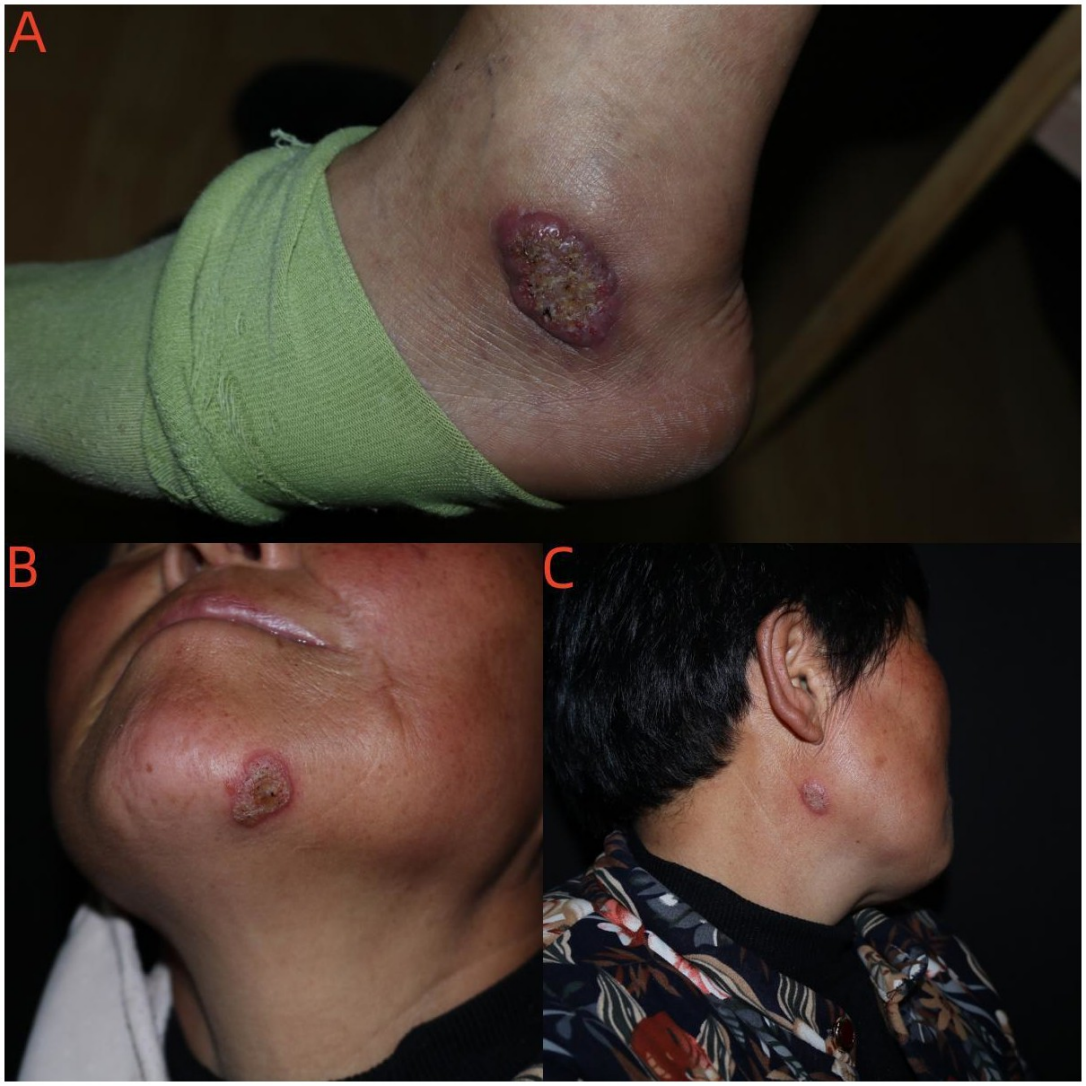
Plaques with ulcers located on the ankle (A) and both sides of the lower jaw (B, C).

In March 2020, a 59-year-old woman with diabetes mellitus presented with a two-year history of ulcerated rashes on the left ankle and both sides of the jaw. Three years prior to the presentation, the patient was diagnosed with type 2 diabetes at a local hospital but had not received standard treatment. The patient worked at a construction site but denied a history of skin trauma. The skin lesions were slightly itchy and sometimes painful. The patient had been assessed at another clinic, where she received surgical treatment for a suspected cutaneous tumor. Physical examination revealed three annular areas of erythematous and raised plaque with an ulcer over the left ankle and both sides of the jaw (Fig 1A–1C).

**Fig 2:**
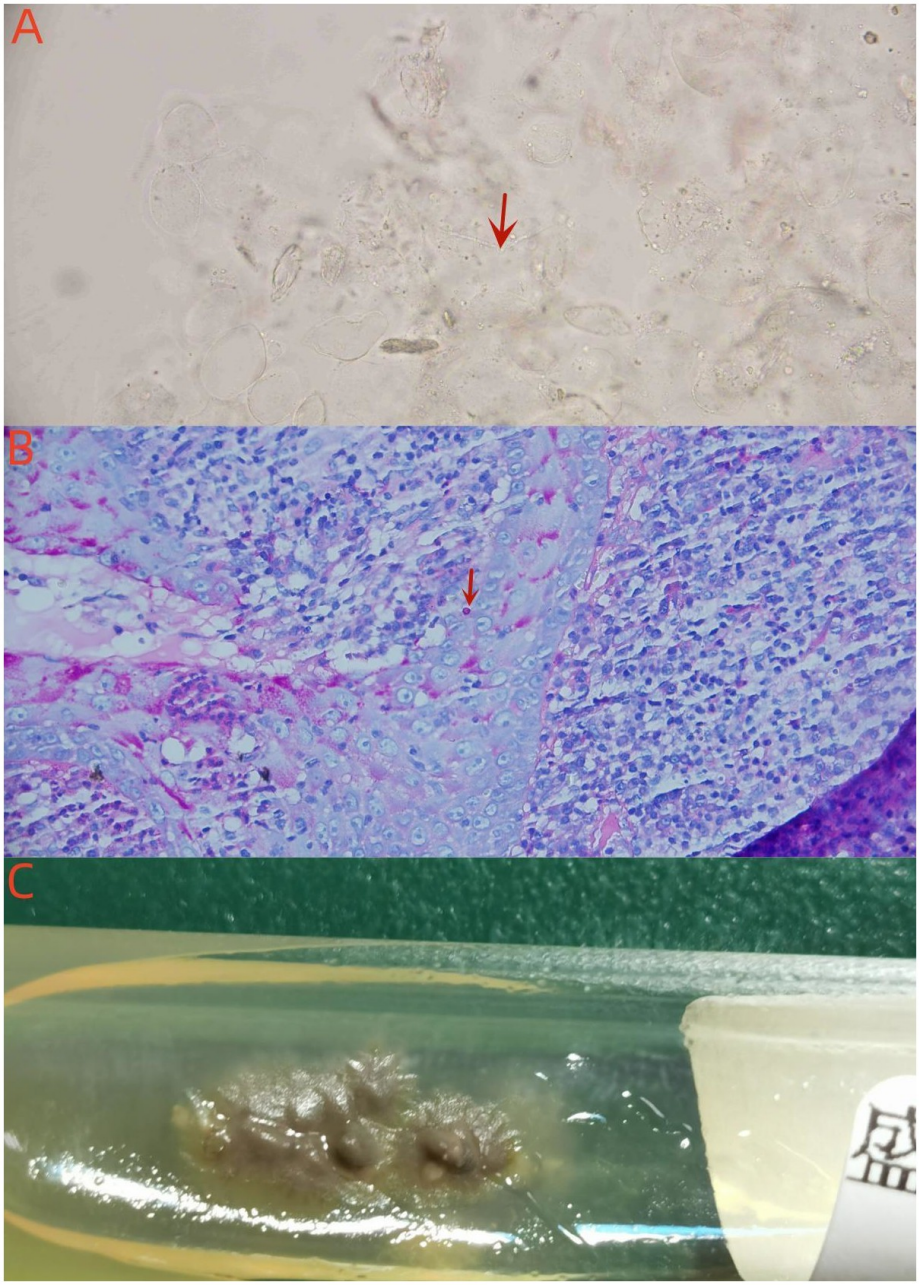
Direct microscopy of exudate smears showing slender hyphae (A, 10% KOH × 400). Periodic acid-Schiff staining showing fungal spores in multinucleated giant cells (B, × 1000). *S*. *globosa* colonies on Sabouraud dextrose agar at 25°C on day 9 (C).

The fasting blood glucose concentration was 10.41 mmol/L (reference range: 3.90–6.10 mmol/L) while the white blood cell count was 3800 cells/mm^3^ (reference range: 4000 to 10000 cells/mm^3^), with 47.7% neutrophils and 30.2% lymphocytes. Liver and renal function tests were done, and the results were within normal ranges. HIV serology was negative. Direct microscopy of exudate smears in either 10% of potassium hydroxide solution (KOH) or fluorescence staining displayed slender hyphae (Fig 2A). Biopsies were taken from skin lesions on the left chin and left ankle, before being immediately sent for histopathological examination and fungal culture. Both lesions showed similar histopathological responses, with pseudoepitheliomatous hyperplasia in the epidermis; neutrophil abscess in the superficial dermis, diffuse lymphocytes, histiocytes, plasma cells, and a few neutrophils infiltrated in the dermis; and multinucleated giant cells.

Periodic acid-Schiff stain revealed dark red spores in multinucleated giant cells (Fig 2B). The mycological culture of exudate from the three lesions and biopsied tissues from the two lesions were performed on Sabouraud dextrose agar that contained 0.05% chloramphenicol, at 25°C. The growth of creamy, brownish-beige colonies that are typical of *Sporothrix* spp. was noted after incubation (Fig 2C). All of the samples developed similar colonies on SDA slant media. Microculture showed thin septate hyphae with conidia in a dense arrangement at its extremities. The fungal culture was confirmed to be *S*. *globosa* by sequencing the ribosomal region ITS1-ITS2 of the rDNA (GenBank accession no. OP614927). Conclusively, the patient was diagnosed with cutaneous disseminated sporotrichosis caused by *S*. *globosa* infection.

Antifungal susceptibility testing using commerical microdilution plate (Trek Diagnostic Systems, Ltd., East Grinstead, United Kingdom) was carried out based on the manufacturer’s instructions. The minimum inhibitory concentration (MIC) values were as follows: anidulafungin (0.25 μg/ml), micafungin (0.5 μg/ml), fluconazole (>256 μg/ml), itraconazole (0.5 μg/ml), voriconazole (>8 μg/ml), caspofungin (1 μg/ml), 5-flucytosine (4.0 μg/ml), posaconazole (1.0 μg/ml) and amphotericin B (<0.12 μg/ml).

**Fig 3:**
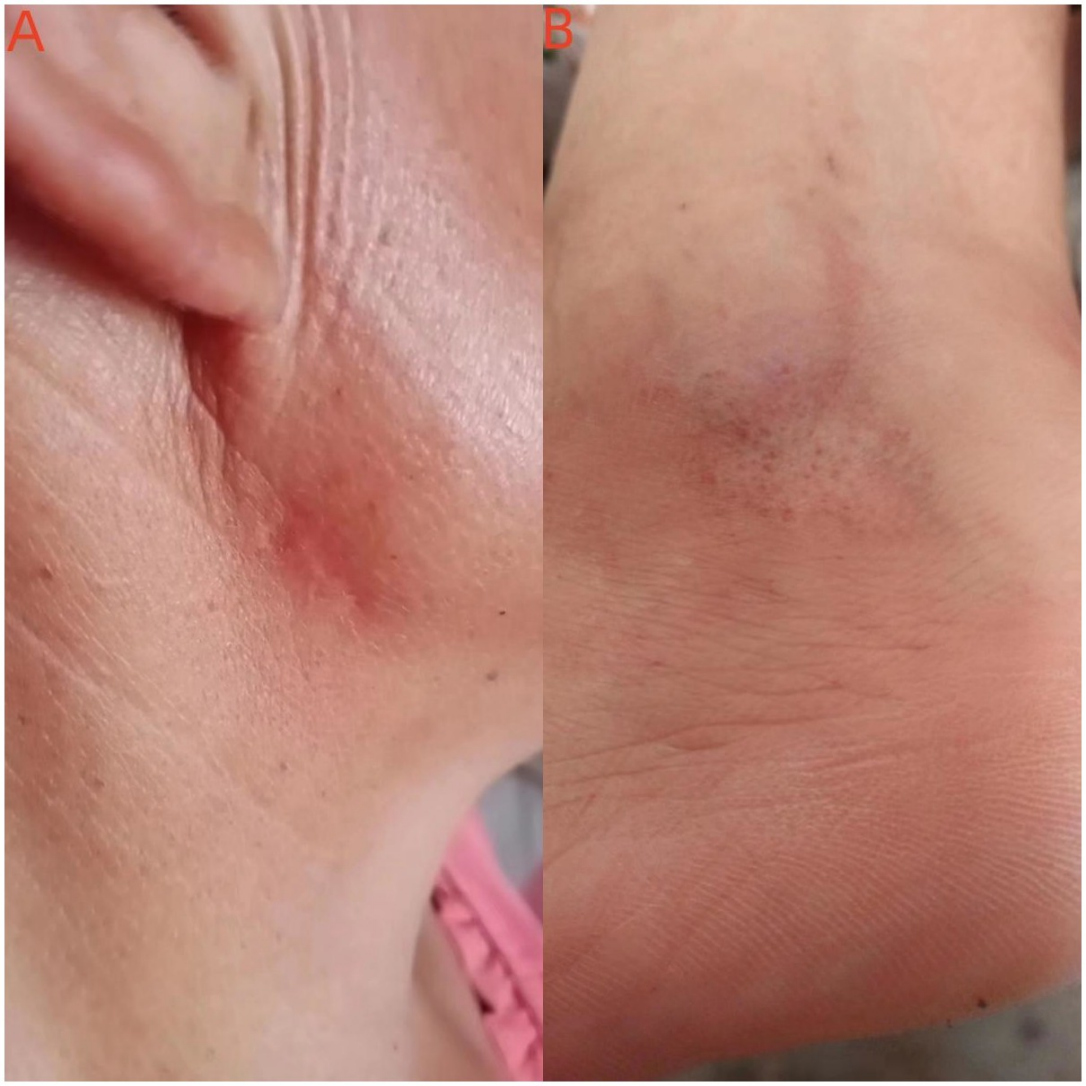
The skin lesions (A, the left ankle; B, the right jaw) were disappeared at 3-year follow-up.

Considering the clinical epidemiological diagnosis on the patient, therapy with itraconazole was initiated at 400 mg/day, for two months. During treatment, liver and kidney function were normal; blood glucose concentration varied between 9.85 mmol/l and 12.57 mmol/l; and the WBC count varied from 3400 cells/mm^3^ to 3700 cells/mm^3^. The patient declined diabetes treatment and tests for identifying the cause of the low count of white blood cells. On the 40^th^ day, Leucogen Tablets (60 mg/day) were administered to the patient to raise the white blood cell count. Finally, after a two-month course of treatment, the lesions resolved, and the WBC count rose to 4100 cells/mm^3^. There was no recurrence after three years of follow-up (Fig 3A and 3B) and the patient is currently being treated for diabetes at a local hospital.

## Methods

### Ethics statement

This study was approved by the ethics committee of Hangzhou Third People’s Hospital and the study participant was informed about the study procedures and written informed consent was obtained.

A systematic literature search cases of sporotrichosis in patients with diabetes has been herein conducted on March 2023, by sourcing both National Library of Medicine (NLM) resources through PubMed, and Scopus and Web of Science, using the following keywords: (“Sporotrichosis”) AND (“diabetes” OR “case report). We first screened the resources, read the titles and abstracts of the articles, and removed the duplicate content, and finally selected 33 items. Then we analyzed the articles according to the inclusion criteria, as topic on sporotrichosis associated with diabetes, English language studies, case reports or research articles, and exclusion criteria, as no risk factor studies or accompanied by other major underlying diseases, resulting in the selection of 16 items. The article details included in the review are reported in Table 1.

**Table 1:** Cases of sporotrichosis in patients with diabetes as reported in scientific literature until March 2023.

Case	Country	Age (years), sex	Presentation	Treatment/Course or total dose	Outcome	Reference
1	Spain	72, male	CDS	ITR/5 months	Recovered	16
2	Peru	67, male	F	SSKI/2 months	Recovered	58
3	Mexico	78, male	Fungemia	Untreated/NA	Died	59
4	Brazil	55, female	Disseminated	AmB/2.5 g	Recovered	60
5	Japan	53, male	Pulmonary	AmB/Unknown	Unknown	61
6	America	23, male	Pulmonary	AmB/100 mg	Unknown	62
7	China	59, male	LC	ITR/10 months	Recovered	63
8	China	60, male	LC	ITR/6 months	Recovered	63
9	Korean	70, male	F	ITR/2 months	Improved	64
10	America	67, male	LC	ITR/6 weeks	Recovered	65
11	America	27, male	Ocular	AmB/215 mg	Recovered	66
12	Italy	64, male	LC	ITR/8 months	Recovered	27
13	Mexico	40, male	LC	ITR + cryosurgery session/6 weeks	Recovered	67
14	Mexico	62, female	F	ITR + cryosurgery session/8 weeks	Recovered	67
15	America	89, male	Disseminated	Unknown	Unknown	68
16	America	58, male	Fungemia	ITR/3 months	Recovered	69

CDS, cutaneous disseminated sporotrichosis; F, fixed sporotrichosis; LC, lymphocutanous sporotrichosis; ITR, itraconazole; SSKI, saturated solution of potassium iodide; AmB, amphotericin B; KCZ, ketoconazole

## Results

In total, 14 articles with 16 patients were identified according to a literature search. Overall, the number of reported cases remains single digit. The detailed characteristics of the 16 cases of sporotrichosis in patients with diabetes are presented in Table 1. There were 14 male and 2 female patients, with median age was 59 (range, 23–89) years. Of the 16 patients, 9 presented with cutaneous form and 7 presented with extracutaneous form. Among the 15 patients who received antifungal therapy, 1 died and the others had a good prognosis.

## Discussion

Sporotrichosis is a subacute/chronic fungal disease that is caused by the dimorphic fungus of the genus *Sporothrix*. This fungal disease can affect both humans and livestock [7]. At present, the consensus is that the S. *schenckii* complex is composed of *S*. *brasiliensis*, *S*. *Schenckii*, *S*. *globosa*, *S*. *mexicana*, and *S*. *luiriei* [8–11]. *S*. *globosa* and *S*. *schenckii* are globally distributed, with the former serving as the primary pathogen for human sporotrichosis in China [12]. *S*. *globosa* is found in decomposing plants, soil, and mosses in various parts of the world. It’s optimum conditions for growth are 22°C and 27°C (71° F and 80° F) and 90% humidity. *S*. *globosa* requires cellulose-rich soil, and a pH level between 3.5 and 9.4 [13].

The infection route is predominantly by traumatic inoculation with contaminated material. However, most patients forget their history of trauma because wounds are typically mild and usually occur a few weeks earlier. Approximately 10 to 62% of the patients recall the trauma related to infection [14–15]. In this case, although the patient denied a history of trauma, the patient was categorized with susceptible populations due to her involvement in construction work for a long time. There is a probability that inconscient traumatic inoculation might have taken place.

Sporotrichosis manifests as a papule or nodule at the trauma site, within a couple of days after traumatic inoculation. After penetrating the skin, the fungus transforms into the yeast form and may remain localized in the subcutaneous tissue or extend along adjacent lymphatic vessels, constituting the fixed or the lymphocutaneous form, respectively [4]. The fungus may be transmitted via a hematogenous route, although this is rare and is characterized by a cutaneous disseminated form [16].

Most disseminated, visceral, and fungemia forms of sporotrichosis are associated with immunosuppression and studies report less than 5% [3,4,17]. De Beurmann *et al* were the first to describe CDS, the third form of cutaneous sporotrichosis, which is a rare variant with multiple lesions without extracutaneous involvement. In their reports, these researchers suggested that the fungus acted as an opportunist [18]. Since then, single or serial cases of CDS were brought forward, only reported in up to 8% of the total cases of sporotrichosis [3,4]. However, there are some exceptions. For example, a report from Mexico showed 24 out of 174 cases of CDS (13,8%) [19]. Generally, the frequency of CDS in our department is not high. From January 2013 to August 2022, a total of 71 cases of cutaneous sporotrichosis were identified, of which only one case was CDS (1/71, 1.41%) [20].

The most frequent predisposing factor of CDS was HIV-AIDS [3,21–24], usually occurring in patients in stage C of AIDS (CD4+ count <200 cells/ mm^3^) [3,17]. Second, CDS can be caused by uncontrolled diabetes [3,4,25]. Overall, the condition may be attributed to immunological deterioration related to cellular response, especially macrophage dysfunction [3,4,26]. Alterations in skin trophism, as well as in specific and non-specific local defenses are important predisposing factors that make diabetic patients more prone to infections [27]. Our patient had uncontrolled diabetes and leukopenia, a scenario that possibly exposed her to CDS. However, in the case reported in this study, the relationship between diabetes and leukopenia remains unclear. Saygin C *et al*. reported an adult type 1 diabetes case with neutropenia associated with anti-neutrophil cytoplasmic antibodies [28]. Another study found that mild but significant neutropenia preceded and accompanied type I diabetes [29]. More research showed an inverse relationship between white blood cell (WBC) count and insulin tolerance, suggesting that leukocytosis was associated with the development and possibly, the progression of diabetes mellitus [30]. In addition, CDS also has been reported in immunocompromised patients with chronic alcoholism [19,31–33], hematologic cancer (leukemia and lymphomas) [34–36], and malnutrition [3,4]. Those who have undergone steroid treatment [2,3,37], and organ transplantation [38,39] have also been classified as relatively more vulnerable to CDS.

It’s also important to note that there are exceptional cases where immunocompetent patients are affected by CDS [17,40–43]. This suggests that there are multiple factors from the fungus and host that contribute to the clinical evolution of sporotrichosis to benign or severe disease [7]. Different species of the genus *Sporothrix* have varying grades of virulence, mainly based on the role of melanine, glycoproteins, and other cell wall components as markers of virulence. These grades of virulence are based on the capacity of the fungi to evade immune recognition [44] and are recognized by human mononuclear cells [45], inducing an immune response [46]. An immunosuppressant status is an essential factor for cutaneous-disseminated and disseminated sporotrichosis [47]. However, Zhang *et al*. found a 10-bp deletion in the ribosomal NTS region of a strain that was isolated from a CDS patient versus control strains that were obtained from fixed cutaneous sporotrichosis [48]. These results suggested the existence of a more virulent strain that may produce cutaneous-disseminated and disseminated sporotrichosis [3,4,17].

Although there are distinct pathogenesis characteristics for each situation, determining whether the multiple cutaneous sporotrichosis lesions are due to repeated inoculations, hematogenous dissemination, or even self-inoculation is a challenge [4]. Our patient’s occupation probably offered relatively enough opportunities for environmental exposure to pathogenic *Sporothrix* spp. Moreover, the fact that she had diabetes mellitus and leukopenia makes it even more difficult to ascertain the cause of the spread of skin lesions. One study suggested that repeated inoculation was more likely to be predominantly responsible for the clinical presentation in CDS patients [49]. The manifestations of CDS were mainly nodules, papules, necrotic lesions, ulcers, and verrucous plaques located in different sites of the body [3,4,17,19,24,50–52]. It is unclear whether clinical manifestations are related to the route of transmission or not.

The standard criterion for sporotrichosis diagnosis involves isolating the etiologic agent [4]. Direct examination and staining are not useful for diagnosing cutaneous-lymphatic and cutaneous-fixed sporotrichosis, since yeasts are observed only in a low percentage (5%–10%) [47]. However, in our recent series of studies, we found that the direct microscopic positive rate of cutaneous sporotrichosis was as high as 40.91%, with hyphae, budding yeast or spores visible under the microscope [20]. Especially in cases of immunocompromised patients, large clusters of yeast are observed, similar to feline sporotrichosis [53,54]. The presence of slender hyphae under direct microscopic may be attributed to low body surface temperature in the sites of the patient’s open skin lesions. So, in CDS patients, direct microscopy is an important diagnostic procedure, considering the high fungal burden in tissues of immunocompromised patients [21,55]. On the other hand, the presence of numerous yeast cells or hyphae upon conducting direct mycological examination and histopathology may be a red flag for a compromised immune system or a highly invasive pathogen fungus.

Generally, itraconazole is effective against any form of sporotrichosis in immunocompromised patients. The dosage varies from 200 mg daily or twice daily, depending on the clinical presentation [4]. However, the treatment guidelines stipulate that amphotericin B, preferably lipidic at 3–5 mg/kg/day, should be administered for more severe forms of sporotrichosis [55]. If the deoxycholate form is used, the recommended doses are 0.7 to 1 mg/kg/day while the treatment duration varies depending on the response and side effects (mainly renal damage) [56,57]. In this case, the patient adequately responded to treatment with itraconazole. For CDS, the choice of treatment should be focused on the route of transmission, with amphotericin B being the first option in the case of hematogenous spread, while itraconazole is considered in cases of self-inoculation or repeated inoculations.

The risk of severe sporotrichosis primarily depends on the immune status of the patients, and the risk is significantly higher in diabetics, although lower than in AIDS patients, compared to immunocompetent patients. Table 1 indicates a male predominance, with the cutaneous and extracutaneous forms accounting for nine and seven cases, respectively [16,27,58–69]. Itraconazole and amphotericin B (AmB) were the most common antifungal regimens that were prescribed in patients for cutaneous and extracutaneous forms, respectively. This suggests that diabetes is a risk factor for severe sporotrichosis, and clinicians should be aware of the potential for severe clinical forms of sporotrichosis in all people with possibly compromised immune function. Invasive fungal disease is a life-threatening complication in type 2 diabetes mellitus patients. *C*. *albicans*, *C*. *neoformans*, and *A*. *fumigatus* are the leading agents. Prolonged hyperglycemia results in unfavorable outcomes. Correction of anemia and hypoalbuminemia might improve prognosis [70]. Similarly, for the treatment of severe sporotrichosis, the control of other underlying diseases such as diabetes is a necessary measure.

## References

[1] Ramos-e-Silva M, Vasconcelos C, Carneiro S, Cestari T. Sporotrichosis. Clin Dermatol. 2007 Mar-Apr;25(2):181–7. doi: 10.1016/j.clindermatol.2006.05.006 17350497

[2] Schechtman RC. Sporotrichosis: Part I. Skinmed. 2010 Jul-Aug;8(4):216–20; quiz 221. 21137607

[3] Bonifaz A, Vázquez-González D. Sporotrichosis: an update. G Ital Dermatol Venereol. 2010 Oct;145(5):659–73. 20930700

[4] Barros MB, de Almeida Paes R, Schubach AO. Sporothrix schenckii and Sporotrichosis. Clin Microbiol Rev. 2011 Oct;24(4):633–54. doi: 10.1128/CMR.00007-11 21976602 PMC3194828

[5] Chakrabarti A, Bonifaz A, Gutierrez-Galhardo MC, Mochizuki T, Li S. Global epidemiology of sporotrichosis. Med Mycol. 2015 Jan;53(1):3–14. doi: 10.1093/mmy/myu062 25526781

[6] Barros MB, Schubach AO, Schubach TM, Wanke B, Lambert-Passos SR. An epidemic of sporotrichosis in Rio de Janeiro, Brazil: epidemiological aspects of a series of cases. Epidemiol Infect. 2008 Sep;136(9):1192–6. doi: 10.1017/S0950268807009727 18028580 PMC2870916

[7] Conceição-Silva F, Morgado FN. Immunopathogenesis of Human Sporotrichosis: What We Already Know. J Fungi (Basel). 2018 Jul 31;4(3):89. doi: 10.3390/jof4030089 30065160 PMC6162489

[8] Oliveira MM, Almeida-Paes R, Gutierrez-Galhardo MC, Zancope-Oliveira RM. Molecular identification of the Sporothrix schenckii complex. Rev Iberoam Micol. 2014 Jan-Mar;31(1):2–6. doi: 10.1016/j.riam.2013.09.008 24270070

[9] Oliveira MM, Almeida-Paes R, Muniz MM, Gutierrez-Galhardo MC, Zancope-Oliveira RM. Phenotypic and molecular identification of Sporothrix isolates from an epidemic area of sporotrichosis in Brazil. Mycopathologia. 2011 Oct;172(4):257–67. doi: 10.1007/s11046-011-9437-3 21701792

[10] Rodrigues AM, de Hoog S, de Camargo ZP. Emergence of pathogenicity in the Sporothrix schenckii complex. Med Mycol. 2013 May;51(4):405–12. doi: 10.3109/13693786.2012.719648 22989196

[11] Rodrigues AM, de Hoog GS, de Cássia Pires D, Brihante RS, Sidrim JJ, Gadelha MF, et al. Genetic diversity and antifungal susceptibility profiles in causative agents of sporotrichosis. BMC Infect Dis. 2014 Apr 23;14:219. doi: 10.1186/1471-2334-14-219 24755107 PMC4021050

[12] Yu X, Wan Z, Zhang Z, Li F, Li R, Liu X. Phenotypic and molecular identification of Sporothrix isolates of clinical origin in Northeast China. Mycopathologia. 2013;176(1–2):67–74. doi: 10.1007/s11046-013-9668-6 23771481 PMC3731519

[13] Charles K, Lowe L, Shuman E, Cha KB. Painful linear ulcers: a case of cutaneous sporotrichosis mimicking pyoderma gangrenosum. JAAD Case Rep. 2017;3:519–21. doi: 10.1016/j.jdcr.2017.07.014 29264381 PMC5729013

[14] Mahajan VK, Sharma NL, Sharma RC, Gupta ML, Garg G, Kanga AK. Cutaneous sporotrichosis in Himachal Pradesh, India. Mycoses. 2005;48(1):25–31. doi: 10.1111/j.1439-0507.2004.01058.x 15679662

[15] Morris-Jones R. Sporotrichosis. Clin Exp Dermatol. 2002 Sep;27(6):427–31. doi: 10.1046/j.1365-2230.2002.01087.x 12372075

[16] Stalkup JR, Bell K, Rosen T. Disseminated cutaneous sporotrichosis treated with itraconazole. Cutis. 2002 May;69(5):371–4. 12041817

[17] Freitas DF, Santos SS, Almeida-Paes R, de Oliveira MM, do Valle AC, Gutierrez-Galhardo MC, et al. Increase in virulence of Sporothrix brasiliensis over five years in a patient with chronic disseminated sporotrichosis. Virulence. 2015;6(2):112–20. doi: 10.1080/21505594.2015.1014274 25668479 PMC4601271

[18] Aram H. Sporotrichosis. A historical approach. Int J Dermatol. 1986 Apr;25(3):203–5. 3516899 10.1111/j.1365-4362.1986.tb02221.x

[19] Bonifaz A, Tirado-Sánchez A, Paredes-Solís V, Cepeda-Valdés R, González GM, Treviño-Rangel RJ, et al. Cutaneous disseminated sporotrichosis: clinical experience of 24 cases. J Eur Acad Dermatol Venereol. 2018 Feb;32(2):e77–e79. doi: 10.1111/jdv.14533 28833587

[20] Zhu H, Xia X, Zhi H, Shen H, Lv W, Sang B, et al. Retrospective analysis of 71 patients with cutaneous sporotrichosis. Mycoses. 2023 Jul;66(7):621–631. doi: 10.1111/myc.13588 37035906

[21] de Oliveira-Esteves ICMR, Almeida Rosa da Silva G, Eyer-Silva WA, Basílio-de-Oliveira RP, de Araujo LF, Martins CJ, et al. Rapidly Progressive Disseminated Sporotrichosis as the First Presentation of HIV Infection in a Patient with a Very Low CD4 Cell Count. Case Rep Infect Dis. 2017;2017:4713140. doi: 10.1155/2017/4713140 29147593 PMC5632852

[22] Freitas DF, de Siqueira Hoagland B, do Valle AC, Fraga BB, de Barros MB, de Oliveira Schubach A, et al. Sporotrichosis in HIV-infected patients: report of 21 cases of endemic sporotrichosis in Rio de Janeiro, Brazil. Med Mycol. 2012 Feb;50(2):170–8. doi: 10.3109/13693786.2011.596288 21859385

[23] Freitas DF, Valle AC, da Silva MB, Campos DP, Lyra MR, de Souza RV, et al. Sporotrichosis: an emerging neglected opportunistic infection in HIV-infected patients in Rio de Janeiro, Brazil. PLoS Negl Trop Dis. 2014 Aug 28;8(8):e3110. doi: 10.1371/journal.pntd.0003110 25166475 PMC4148221

[24] Moreira JA, Freitas DF, Lamas CC. The impact of sporotrichosis in HIV-infected patients: a systematic review. Infection. 2015 Jun;43(3):267–76. doi: 10.1007/s15010-015-0746-1 25701221

[25] Solorzano S, Ramirez R, Cabada MM, Montoya M, Cazorla E. Esporotricosis cutánea diseminada con compromiso articular en una mujer con diabetes tipo 2 [Disseminated cutaneous sporotrichosis with joint involvement in a woman with type 2 diabetes]. Rev Peru Med Exp Salud Publica. 2015 Jan-Mar;32(1):187–90. Spanish. 26102124

[26] Song Y, Li SS, Zhong SX, Liu YY, Yao L, Huo SS. Report of 457 sporotrichosis cases from Jilin province, northeast China, a serious endemic region. J Eur Acad Dermatol Venereol. 2013 Mar;27(3):313–8. doi: 10.1111/j.1468-3083.2011.04389.x 22176524

[27] Lembo S, Cirillo T, Marasca C, Lo Conte V, Lembo C, Balato A, et al. Sporotrichosis: long-term treatment and follow-up in a diabetic patient from Southern Italy. G Ital Dermatol Venereol. 2016 Oct;151(5):576–8. 27595209

[28] Saygin C, Uzunaslan D, Ugurlu S. Antineutrophil cytoplasmic antibody-associated neutropenia in a patient with type 1 diabetes mellitus. Laboratory hematology: official publication of the International Society for Laboratory Hematology. 2014; 20:7–8. doi: 10.1532/LH96.13001 25000946

[29] Valle A, Giamporcaro GM, Scavini M, Stabilini A, Grogan P, Bianconi E, et al. Reduction of circulating neutrophils precedes and accompanies type 1 diabetes. Diabetes. 2013; 62(6):2072–7. doi: 10.2337/db12-1345 23349491 PMC3661622

[30] Vozarova B, Weyer C, Lindsay RS, Pratley RE, Bogardus C, Tataranni PA. High white blood cell count is associated with a worsening of insulin sensitivity and predicts the development of type 2 diabetes. Diabetes. 2002; 51(2):455–61. doi: 10.2337/diabetes.51.2.455 11812755

[31] Ramírez Soto MC. Sporotrichosis: The Story of an Endemic Region in Peru over 28 Years (1985 to 2012). PLoS One. 2015 Jun 1;10(6):e0127924. doi: 10.1371/journal.pone.0127924 26030742 PMC4452310

[32] Espinoza-Hernández CJ, Jesús-Silva A, Toussaint-Caire S, Arenas R. Disseminated sporotrichosis with cutaneous and testicular involvement. Actas Dermosifiliogr. 2014 Mar;105(2):204–6. English, Spanish. doi: 10.1016/j.ad.2013.02.002 23746386

[33] Nassif PW, Granado IR, Ferraz JS, Souza R, Nassif AE. Esporotricose cutânea de apresentação atípica em paciente etilista [Atypical presentation of cutaneous sporotrichosis in an alcoholic patient]. Dermatol Online J. 2012 Jun 15;18(6):12. Portuguese. 22747936

[34] Ewing GE, Bosl GJ, Peterson PK. Sporothrix schenckii meningitis in a farmer with Hodgkin’s disease. Am J Med. 1980 Mar;68(3):455–7. doi: 10.1016/0002-9343(80)90120-5 7361812

[35] Kumar S, Kumar D, Gourley WK, Alperin JB. Sporotrichosis as a presenting manifestation of hairy cell leukemia. Am J Hematol. 1994 Jun;46(2):134–7. doi: 10.1002/ajh.2830460214 8172180

[36] Bunce PE, Yang L, Chun S, Zhang SX, Trinkaus MA, Matukas LM. Disseminated sporotrichosis in a patient with hairy cell leukemia treated with amphotericin B and posaconazole. Med Mycol. 2012 Feb;50(2):197–201. doi: 10.3109/13693786.2011.584074 21612561

[37] Severo LC, Festugato M, Bernardi C, Londero AT. Widespread cutaneous lesions due to Sporothrix schenckii in a patient under a long-term steroids therapy. Rev Inst Med Trop Sao Paulo. 1999 Jan-Feb;41(1):59–62. doi: 10.1590/s0036-46651999000100010 10436671

[38] Gewehr P, Jung B, Aquino V, Manfro RC, Spuldaro F, Rosa RG, et al. Sporotrichosis in renal transplant patients. Can J Infect Dis Med Microbiol. 2013 Summer;24(2):e47–9. doi: 10.1155/2013/603405 24421819 PMC3720015

[39] Gullberg RM, Quintanilla A, Levin ML, Williams J, Phair JP. Sporotrichosis: recurrent cutaneous, articular, and central nervous system infection in a renal transplant recipient. Rev Infect Dis. 1987 Mar-Apr;9(2):369–75. doi: 10.1093/clinids/9.2.369 3296101

[40] Ferreira CP, do Valle AC, Freitas DF, Reis R, Galhardo MC. Pregnancy during a sporotrichosis epidemic in Rio de Janeiro, Brazil. Int J Gynaecol Obstet. 2012 Jun;117(3):294–5. doi: 10.1016/j.ijgo.2012.02.003 22445393

[41] Yap F.B. Disseminated cutaneous sporotrichosis in an immunocompetent individual. Int. J. Infect. Dis. 2011, 15, 727–729. 10.1016/j.ijid.2011.05.005 21719337

[42] omero-Cabello R, Bonifaz A, Romero-Feregrino R, Sánchez CJ, Linares Y, Zavala JT, et al. Disseminated sporotrichosis. BMJ Case Rep. 2011 Mar 25;2011:bcr1020103404. 10.1136/bcr.10.2010.3404 PMC3070371 22700076

[43] Hassan K, Turker T, Zangeneh T. Disseminated sporotrichosis in an immunocompetent patient. Case Reports Plast Surg Hand Surg. 2016 May 31;3(1):44–7. doi: 10.3109/23320885.2016.1168703 27583270 PMC4996056

[44] Hernández-Chávez MJ, Pérez-García LA, Niño-Vega GA, Mora-Montes HM. Fungal Strategies to Evade the Host Immune Recognition. J Fungi (Basel). 2017 Sep 23;3(4):51. doi: 10.3390/jof3040051 29371567 PMC5753153

[45] Martínez-Álvarez JA, Pérez-García LA, Mellado-Mojica E, López MG, Martínez-Duncker I, Lópes-Bezerra LM, et al. Sporothrix schenckii sensu stricto and Sporothrix brasiliensis Are Differentially Recognized by Human Peripheral Blood Mononuclear Cells. Front Microbiol. 2017 May 10;8:843. doi: 10.3389/fmicb.2017.00843 28539922 PMC5423980

[46] Alba-Fierro CA, Pérez-Torres A, López-Romero E, Cuéllar-Cruz M, Ruiz-Baca E. Cell wall proteins of Sporothrix schenckii as immunoprotective agents. Rev Iberoam Micol. 2014 Jan-Mar;31(1):86–9. doi: 10.1016/j.riam.2013.09.017 24257472

[47] Bonifaz A, Tirado-Sánchez A. Cutaneous Disseminated and Extracutaneous Sporotrichosis: Current Status of a Complex Disease. J Fungi (Basel). 2017 Feb 10;3(1):6. doi: 10.3390/jof3010006 29371525 PMC5715962

[48] Zhang Z, Liu X, Lv X, Lin J. Variation in genotype and higher virulence of a strain of Sporothrix schenckii causing disseminated cutaneous sporotrichosis. Mycopathologia. 2011 Dec;172(6):439–46. doi: 10.1007/s11046-011-9441-7 21717135

[49] de Lima Barros MB, de Oliveira Schubach A, Galhardo MC, Schubach TM, dos Reis RS, Conceição MJ, et al. Sporotrichosis with widespread cutaneous lesions: report of 24 cases related to transmission by domestic cats in Rio de Janeiro, Brazil. Int J Dermatol. 2003 Sep;42(9):677–81. doi: 10.1046/j.1365-4362.2003.01813.x 12956676

[50] Shelley WB, Sica PA Jr. Disseminated sporotrichosis of the skin and bone cured with 5-fluorocytosine. J Am Acad Dermatol. 1983;8:229–235. 6826816 10.1016/s0190-9622(83)70029-0

[51] Stroud JD. Sporotrichosis presenting as pyoderma gangrenosum. Arch Dermatol. 1968 Jun;97(6):667–70. 5652972

[52] Ticoras CJ, Schroeter AL, Hornbeck KL. Disseminated ulcerated papules and nodules. Cutaneous disseminated sporotrichosis. Arch Dermatol. 1996 Aug;132(8):963–4, 966–7. doi: 10.1001/archderm.1996.03890320113018 8712848

[53] Pereira SA, Gremião ID, Kitada AA, Boechat JS, Viana PG, Schubach TM. The epidemiological scenario of feline sporotrichosis in Rio de Janeiro, State of Rio de Janeiro, Brazil. Rev Soc Bras Med Trop. 2014 May-Jun;47(3):392–3. doi: 10.1590/0037-8682-0092-2013 25075494

[54] Sanchotene KO, Madrid IM, Klafke GB, Bergamashi M, Della Terra PP, Rodrigues AM, et al. Sporothrix brasiliensis outbreaks and the rapid emergence of feline sporotrichosis. Mycoses. 2015 Nov;58(11):652–8. doi: 10.1111/myc.12414 26404561

[55] Schechtman RC, Crignis GS, Pockstaller MP, Azulay-Abulafia L, Quintella LP, Belo M. Molluscum-like lesions in a patient with sporotrichosis. An Bras Dermatol. 2011 Nov-Dec;86(6):1217–9. English, Portuguese. doi: 10.1590/s0365-05962011000600028 22281918

[56] Kauffman CA, Bustamante B, Chapman SW, Pappas PG; Infectious Diseases Society of America. Clinical practice guidelines for the management of sporotrichosis: 2007 update by the Infectious Diseases Society of America. Clin Infect Dis. 2007 Nov 15;45(10):1255–65. doi: 10.1086/522765 17968818

[57] Mahajan VK. Sporotrichosis: an overview and therapeutic options. Dermatol Res Pract. 2014;2014:272376. doi: 10.1155/2014/272376 25614735 PMC4295339

[58] Ramírez-Soto M, Lizárraga-Trujillo J. Esporotricosis granulomatosa: presentación de dos casos inusuales [Granulomatous sporotrichosis: report of two unusual cases]. Rev Chilena Infectol. 2013 Oct;30(5):548–53. 24248171 10.4067/S0716-10182013000500013

[59] Castrejón OV, Robles M, Zubieta Arroyo OE. Fatal fungaemia due to Sporothrix schenckii. Mycoses. 1995 Sep-Oct;38(9–10):373–6. doi: 10.1111/j.1439-0507.1995.tb00067.x 8569812

[60] Kohler LM, Hamdan JS, Ferrari TC. Successful treatment of a disseminated Sporothrix schenckii infection and in vitro analysis for antifungal susceptibility testing. Diagn Microbiol Infect Dis. 2007 May;58(1):117–20. doi: 10.1016/j.diagmicrobio.2006.11.002 17498572

[61] Nakano H, Suzuki A, Tojima H, Imahashi M, Kashiyama T, Honda A, et al. [A case of primary pulmonary sporotrichosis]. Nihon Kyobu Shikkan Gakkai Zasshi. 1989 Feb;27(2):220–4. 2747077

[62] Pueringer RJ, Iber C, Deike MA, Davies SF. Spontaneous remission of extensive pulmonary sporotrichosis. Ann Intern Med. 1986 Mar;104(3):366–7. doi: 10.7326/0003-4819-104-3-366 3946980

[63] Zhang Y, Hagen F, Wan Z, Liu Y, Liu Y, Wang Q, et al. Two cases of sporotrichosis of the right upper extremity in right-handed patients with diabetes mellitus. Rev Iberoam Micol. 2016 Jan-Mar;33(1):38–42. doi: 10.1016/j.riam.2015.02.001 25982353

[64] Mohamad N, Badrin S, Wan Abdullah WNH. A Diabetic Elderly Man with Finger Ulcer. Korean J Fam Med. 2018 Mar;39(2):126–129. doi: 10.4082/kjfm.2018.39.2.126 29629046 PMC5876048

[65] Milby AH, Pappas ND, O’Donnell J, Bozentka DJ. Sporotrichosis of the upper extremity. Orthopedics. 2010 Apr;33(4). doi: 10.3928/01477447-20100225-27 20415315

[66] Agger WA, Caplan RH, Maki DG. Ocular sporotrichosis mimicking mucormycosis in a diabetic. Ann Ophthalmol. 1978 Jun;10(6):767–71. 307934

[67] Robles-Tenorio A, Rocha-Mendez LE, Tarango-Martinez VM. Cryosurgery as adjuvant treatment for cutaneous sporotrichosis in two patients with diabetes mellitus type 2. Clin Exp Dermatol. 2023 Mar 1;48(3):257–259. doi: 10.1093/ced/llac090 36763769

[68] Aronowitz PB, Gilroy M, Christiansen KN. Disseminated Sporotrichosis with Osteolytic Bone Involvement. J Gen Intern Med. 2017 Sep;32(9):1063. doi: 10.1007/s11606-017-4048-4 28349410 PMC5570735

[69] Baker JH, Goodpasture HC, Kuhns HR Jr, Rinaldi MG. Fungemia caused by an amphotericin B-resistant isolate of Sporothrix schenckii. Successful treatment with itraconazole. Arch Pathol Lab Med. 1989 Nov;113(11):1279–81. 2554844

[70] Lao M, Li C, Li J, Chen D, Ding M, Gong Y. Opportunistic invasive fungal disease in patients with type 2 diabetes mellitus from Southern China: Clinical features and associated factors. J Diabetes Investig. 2020 May;11(3):731–744. 10.1111/jdi.13183 PMC7232281 31758642

