# Supplementary material for: Cutaneous disseminated sporotrichosis associated with diabetes: A case report and literature review
Source: PLoS Negl Trop Dis. 2023 Sep 18;17(9):e0011647. doi: 10.1371/journal.pntd.0011647 (PMC10538719; doi:10.1371/journal.pntd.0011647)
Supplement: Attachment — Submitted filename: response(R1).docx [file pntd.0011647.s001.docx]

Dear Editor and Reviewers:

Many thanks for the hard work of the Editor and Reviewers.

The comments of the reviewers and editor were highly insightful and enabled us to greatly improve the quality of our manuscript. We have tried our best to modify the manuscript, we hope to meet with the requirements of your journal. Some mistakes in the main text were corrected. Now, please see below for our point-by-point response to the reviewers' comments.

**Methods**

Reviewer #1: The structure and methodology of the case is adequate

**Response:** Many thanks for the reviewer^’^s positive comments.

Reviewer #2: Actually, the article format of this MS is case report instead of research article, so I suggest that this submission is more suited as case report format.

Besides, could the author offer the figure of direct microscopy, because it was stated that direct microscopy displayed slender hyphae. Moreover, it was stated that the patient healed after two months' therapy, but there are not any figures after healed.

**Response:** Many thanks for the reviewer’s suggestions. We attempted to fully analyze the epidemiological characteristics of CDS in diabetic patients through the presentation of this case and literature review. Case report will be not sufficient for our purposes, we shall be very honored if this arrangement is approved.

This is a good suggestion, we have added the figure of direct microscopy and figures after healed. Thanks!

Reviewer #3: The draft manuscript “Disseminated cutaneous sporotrichosis (CDS) refers to the case of a woman with diabetes mellitus associated with CDS”. Based on the laboratory findings, there was an increase in blood glucose and a decrease in the leukocyte count, standard for mycological diagnosis and confirmed by DNA sequencing. The study adequately described antifungal therapy. The paper can be accepted, but be interesting to adapt it to the suggestions below.

**Response:** Many thanks for the reviewer’s positive comments.

**Results**

Reviewer #1: The results of the case are adequate

**Response:** Many thanks for the reviewer’s positive comments.

Reviewer #2: Reviewer #2: The MS reviewed the literatures of CDS, but only cited the table in the results of male predominance. The table could review the data of underling disease and the duration of treatment.

Besides, the MS stated that the risk factor for CDS is diabetes but did not show the results of high-throughput genome sequencing or test for HIV.

**Response:** Many thanks for the reviewer’s suggests. Table 1 shows the various cases of sporotrichosis with diabetes. We have added the course of treatment in table 1.

The patient's serum was HIV-negative, which we have added in the manuscript.

Reviewer #3: The authors reported the results of the leukogram as its monitoring during the treatment, thus verifying the importance of the procedure but not the information on the red blood cells. In these patients, anemia and hypoalbuminemia may occur in parallel. Add the information, if possible, and insert the reference below.

Lao et al. (2020) found that invasive fungal disease in patients with type 2 diabetes mellitus does not have a good prognosis, with complications potentially fatal, as prolonged hyperglycemia results in unfavorable outcomes. In these patients, anemia and hypoalbuminemia occur in association, and their correction helps to improve the prognosis.

Lao et al. (2020) mention the relationship between diabetes and leukopenia, even if they do not observe its significance. The fact that the clinical improvement of the reported patient is favorable with the improvement of his leukogram suggests future investigations.

The authors mention that the patient had uncontrolled diabetes. It is relevant to report whether it is type 1 or type 2 diabetes and whether the treatment is adequate for your underlying disease.

**Response:** The reviews were highly professionaand, we were very inspiredl. Laboratory tests showed that the patient's red blood cells and serum albumin were normal. Thanks a lot!

This paper (Lao et al. 2020) is very valuable for reference, and we have quoted some of the views in the manuscript.

The patient has been diagnosed with type 2 diabetes and is currently receiving standard treatment at a local hospital. We have made relevant statements in the manuscript.

**Conclusions**

Reviewer #1: The conclusions reached are correct.

**Response:** Many thanks for the reviewer’s positive comments.

Reviewer #2: The MS stated that the risk factor for CDS is diabetes but did not show the results of high-throughput genome sequencing or test for HIV.

**Response:** The patient was HIV negative, as we have stated in the manuscript. Thanks!

Reviewer #3: The study would be more impactful if the authors added other studies of diabetes and fungal infections to transpose this information into sporotrichosis since is still obscure.

**Response:** Many thanks for the reviewer’s valuable comments. We have included relevant information in the last paragraph of the manuscript.

**Editorial and Data Presentation Modifications?**

Reviewer #1: No

**Response:** NA.

Reviewer #2: (No Response)

**Response:** NA.

Reviewer #3: The study would be more impactful if the authors added other studies of diabetes and fungal infections to transpose this information into sporotrichosis since is still obscure.

**Response:** Many thanks for the reviewer’s valuable comments. We have included relevant information in the last paragraph of the manuscript.

**Summary and General Comments**

Reviewer #1: It is an interesting case that emphasizes how cases of sporotrichosis can spread through the skin.

The only thing that I suggest to the authors that they add in the discussion is that these cases usually present little yeast presence, are similar to lymphangitic cases and that they behave differently when there is immunosuppression because the number of yeasts is greater

**Response:** Many thanks for the reviewer’s insightful comment. Thanks!

This is a good suggestion, and we have added relevant content to the discussion section of the manuscript.

Reviewer #2: (No Response)

**Response:** NA

Reviewer #3: Suggestion of reference

Lao M, Li C, Li J, Chen D, Ding M, Gong Y. Opportunistic invasive fungal disease in patients with type 2 diabetes mellitus from Southern China: Clinical features and associated factors. J Diabetes Investig. 2020 May;11(3):731-744. doi: 10.1111/jdi.13183. Epub 2019 Dec 20. PMID: 31758642; PMCID: PMC7232281.

Format the references according to the journal's rules.

Reference 42 is incorrect.

**Response:** This is a good suggestion, and we cite it in the discussion section of the manuscript.

We have corrected the format of reference 42. Thanks!

Thank you again! We look forward to your reply.

Best wishes!
