# Supplementary material for: Cutaneous disseminated sporotrichosis associated with diabetes: A case report and literature review
Source: PLoS Negl Trop Dis. 2023 Sep 18;17(9):e0011647. doi: 10.1371/journal.pntd.0011647 (PMC10538719; doi:10.1371/journal.pntd.0011647)
Supplement: Attachment — Submitted filename: response(R3).docx [file pntd.0011647.s002.docx]

Dear Editor and Reviewers:

Many thanks for the hard work of the Editor and Reviewers.

Now, please see below for our point-by-point response to the reviewer's comments.

**Reviewer comments:**

-Thank you for the revision.

**Response:** We really appreciate the encouragement of the review expert.

-It is hard to find the slender hyphae in Figure 2A. And as slender hyphae are not commonly seen in direct smear, the author should discuss this finding.

**Response:** Figure 2A is not that high resolution, but we are confident that it is hyphae, as we recently reported (Mycoses. 2023 Jul;66(7):621-631. doi: 10.1111/myc.13588. Epub 2023 Apr 10. PMID: 37035906.), the direct microscopic positive rate of cutaneous sporotrichosis was as high as 40.9%, and various fungal structures could be seen. We have added the corresponding discussion in the manuscript. Thanks!

-It is not clear if all lesions were culture positive. Please address this point.

**Response:** We are sorry we didn't make it clear. We took samples from three skin lesions, as well as tissue from one lower jaw and one ankle for fungal culture, all of which yielded identical colonies. We have made corresponding additions in the manuscript. Thanks a lot!

Thank you again! We look forward to your reply.

Best wishes!
